# Anti-Tumour Promoting Activity and Antioxidant Properties of Girinimbine Isolated from the Stem Bark of *Murraya koenigii *S

**DOI:** 10.3390/molecules17044651

**Published:** 2012-04-20

**Authors:** Yih Yih Kok, Lim Yang Mooi, Kartini Ahmad, Mohd Aspollah Sukari, Nashriyah Mat, Mawardi Rahmani, Abdul Manaf Ali

**Affiliations:** 1 Department of Cell and Molecular Biology, Universiti Putra Malaysia, 43400 UPM Serdang, Selangor, Malaysia; Email: yihyih_kok@imu.edu.my (Y.Y.K.); ymlim@utar.edu.my (L.Y.M.); 2 Department of Human Biology, International Medical University, 126 Jalan Jalil Perkasa 19, Bukit Jalil, 57000 Kuala Lumpur, Malaysia; 3 Department of Preclinical Sciences, Universiti Tunku Abdul Rahman, Lot PT21144, Jalan Sungai Long, 43000 Kajang, Selangor, Malaysia; 4 Department of Chemistry, Universiti Putra Malaysia, 43400 UPM Serdang, Selangor, Malaysia; Email: kartini@fsmt.upsi.edu.my (K.A.); aspollah@science.upm.edu.my (M.A.S.); mawardi@science.upm.edu.my (M.R.); 5 Department of Chemistry, Faculty of Science and Mathematics, Universiti Pendidikan Sultan Idris, 35900 Tanjong Malim, Perak, Malaysia; 6 Faculty of Agriculture and Biotechnology, Universiti Sultan Zainal Abidin, Gong Badak Campus, 21300 Kuala Terengganu, Terengganu, Malaysia; Email: nashriyah@unisza.edu.my (N.M.)

**Keywords:** *Murraya koenigii*, girinimbine, anti-tumour promoting activity, antioxidative, superoxide

## Abstract

Girinimbine, a carbazole alkaloid isolated from the stem bark of *Murraya koenigii *was tested for the *in vitro* anti-tumour promoting and antioxidant activities. Anti-tumour promoting activity was determined by assaying the capability of this compound to inhibit the expression of early antigen of Epstein-Barr virus (EA-EBV) in Raji cells that was induced by the tumour promoter, phorbol 12-myristate 13-acetate. The concentration of this compound that gave an inhibition rate at fifty percent was 6.0 µg/mL and was not cytotoxic to the cells. Immunoblotting analysis of the expression of EA-EBV showed that girinimbine was able to suppress restricted early antigen (EA-R). However, diffused early antigen (EA-D) was partially suppressed when used at 32.0 µg/mL. Girinimbine exhibited a very strong antioxidant activity as compared to α-tocopherol and was able to inhibit superoxide generation in the 12-*O*-tetradecanoylphorbol-13-acetate (TPA)-induced differentiated premyelocytic HL-60 cells more than 95%, when treated with the compound at 5.3 and 26.3 µg/mL, respectively. However girinimbine failed to scavenge the stable diphenyl picryl hydrazyl (DPPH)-free radical.

## 1. Introduction

*Murraya koenigii *is one of the two species of *Murraya* found in Peninsular Malaysia [1]. The plant is known locally as curry leaf due to its aromatic smell. The leaves are used as traditional vegetable (ulam), which help promote appetite and digestion, and provide natural flavouring in curries [2]. Various parts of this plant were used to cure several diseases. For example, the leaves are used to treat diarrhoea, dysentery and digestive disorder [3]. The leaves are also used in Indian traditional medicine to prevent and cure diabetes. It was reported that the leaves inhibit α-amylase and also increased insulin production in animal models [4]. The roots are used to cure piles, inflammation and itching, while powdered leaves are used to aid healing of fresh cuts [5]. The barks and roots are also used to relieve skin eruptions and bites by poisonous animals [6]. The leaves were reported to have a very strong antioxidative activity [7,8] and the methanolic extract of the leaves contain antinematodal activity against the pine wood nematode, *Bursaphelenchus* *xylophilus* [9]. 

**Figure 1:**
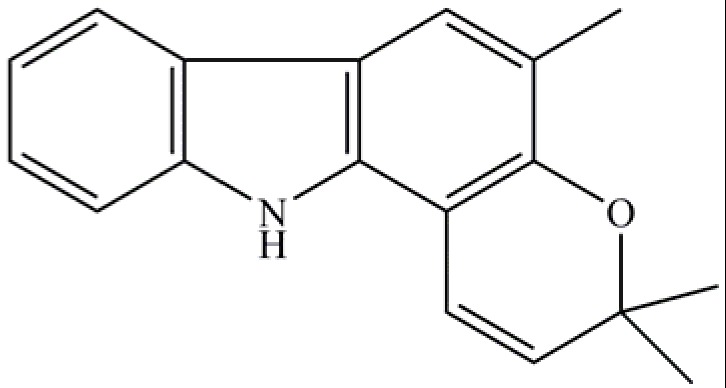
Girinimbine.

*Murraya koenigii *is known to be the richest source of carbazole alkaloids [10,11,12,13]. Girinimbine (Figure 1), together with mahanimbine and murrayanine, were isolated from the barks of stem of *M. koenigii *[13]. Total syntheses through novel synthetic methods of all these carbazole alkaloids which are biologically active have been reported by Knolker and Reddy [14] and Knolker [15]. Furthermore, highly efficient routes to the carbazole girinimbine were also established [16,17]. Ko *et al.* [18] have reported that girinimbine isolated from *M. euchrestifolia* was shown to have anti-platelet activity through the inhibition of cyclooxygenase activity. The compound was reported to be cytotoxic to various cell lines which caused the cells to die through apoptosis cell death [19,20].

In this paper, we report that girinimbine isolated from the stem bark of *M. koenigii* possesses a very strong anti-tumour promoting activity when assayed for the inhibition of early antigen expression of Epstein-Barr virus in Raji cells but compound was not cytotoxic to the cells. This compound was shown to have a strong antioxidant activity as assayed using the ferric thiocyanate method and inhibition of superoxide (O_2_^−^) generation in the TPA-induced promyelocytic HL-60 cells.

## 2. Results and Discussion

**Figure 2:**
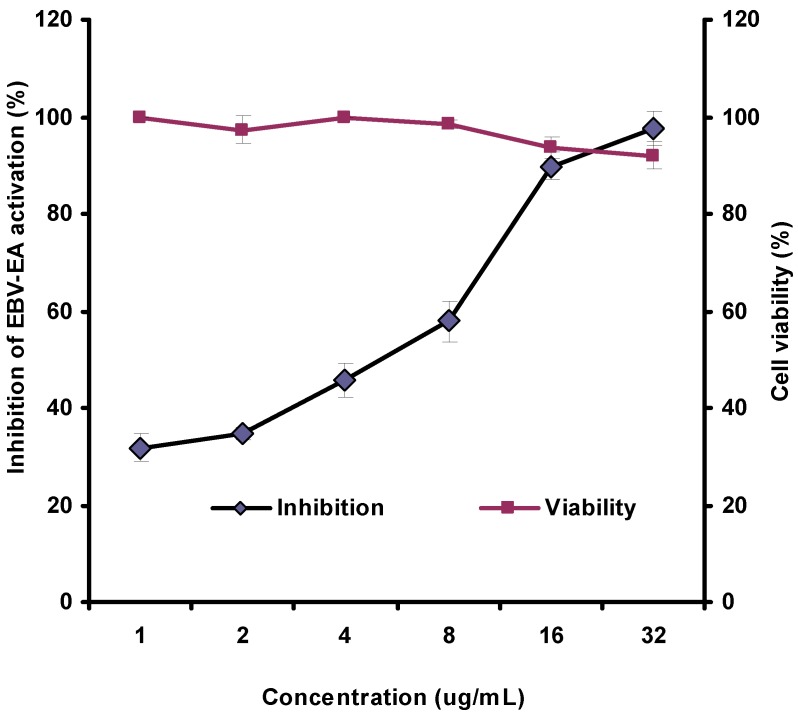
Effect of girinimbine on the early antigen (EA) of Epstein-Barr virus activation and the viability of Raji cells.

The *in vitro* anti-tumour promoting activity of girinimbine was determined by measuring the percentage inhibition of induced early antigen (EA) of EBV on the surface of Raji cells. Raji cells are B-human lymphoblastoids latently infected with EBV, in which the early antigen of the virus can be induced by phorbol 12-myristate 13-acetate and *n*-butyrate to express on the surface of cells which can be detected by imunofluorescence using human antisera of nasopharyngeal carcinoma [21]. In this study, we have shown that girinimbine strongly inhibited the induction of EA of EBV more than 90% when tested at 16.0 and 32.0 µg/mL. The inhibition rate was moderate when tested at 8.0 µg/mL (inhibition rate 58%) and low at 4.0, 2.0 and 1.0 µg/mL (inhibition rates of 46, 35 and 32%, respectively). The inhibition rate at fifty percent of the compound extrapolated from the dose response curve was 6.0 µg/mL (Figure 2). The compound was not cytotoxic to the Raji cells where the viability of the cells was more than 90% at all the concentration tested.

**Figure 3:**
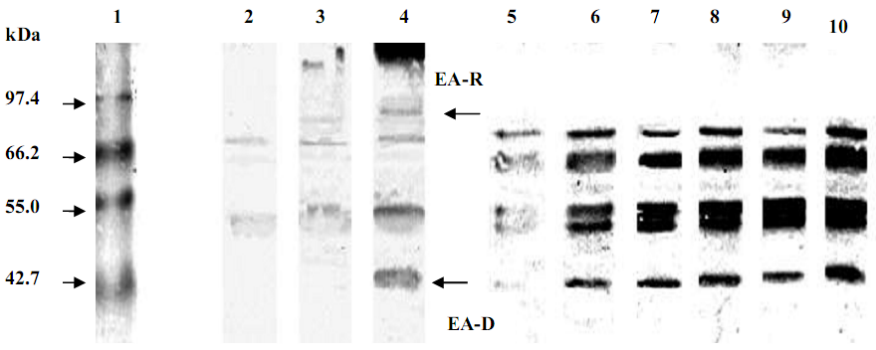
Effects of different concentrations of girinimbine on the synthesis of EBV-early protein diffuse (EA-D) (bottom arrow) and restricted (EA-R) (top arrow) from Raji cells treated with phorbol 12-myristate 13-acetate (PMA, 0.05 µM) and sodium-*n*-butyrate (3 mM) by immunoblotting analysis. Lane 1 was the pre-stained SDS-PAGE standard, mid-range marker. Lane 2 is the untreated Raji cells (C1), Lane 3 is sodium-*n*-butyrate treated Raji cells (C2), Lane 4 is PMA and sodium-*n*-butyrate treated Raji cells (C3), Lanes 5 to 10 are Raji cells treated with girinimbine (1 to 32 µg/mL).

The immunoblotting analysis of EBV-early protein diffused (D) and restricted (R) from Raji cells, induced by PMA and sodium-*n*-butyrate were studied with different concentrations of girinimbine. The EBV-EA classified into diffused type (EA-D), which can be detected both in the nucleus and the cytoplasm as a high intensity 50–52 kDa protein band. The restricted type (EA-R) is only detected in the cytoplasm which can be observed as a less intense 85 kDa protein band [22,23]. Figure 2 shows Raji cells induced with PMA and *n*-butyrate expressed both EA-R and EA-D whereas untreated cells and cells treated only with sodium-*n*-butyrate only expressed EA-R. A similar observation was reported by Ali *et al.* [22] and Kondo *et al.* [23]. In this study, the EA-R was completely suppressed by girinimbine at all concentrations tested (1.0, 2.0, 4.0, 8.0, 16.0 and 32.0 µg/mL). On the other hand, the EA-D was partially suppressed only at a concentration of 32.0 µg/mL (Figure 3). 

The antioxidative activity of girinimbine was measured by the FTC method at a concentration of 0.02% in an aqueous ethanolic solution. The FTC method measures the amount of peroxide produced during initial stages of lipid oxidation. During the oxidation process, peroxide was gradually decomposed to lower molecule compounds that gave red colour, which can be measured at 500 nm wavelength. 

**Figure 4:**
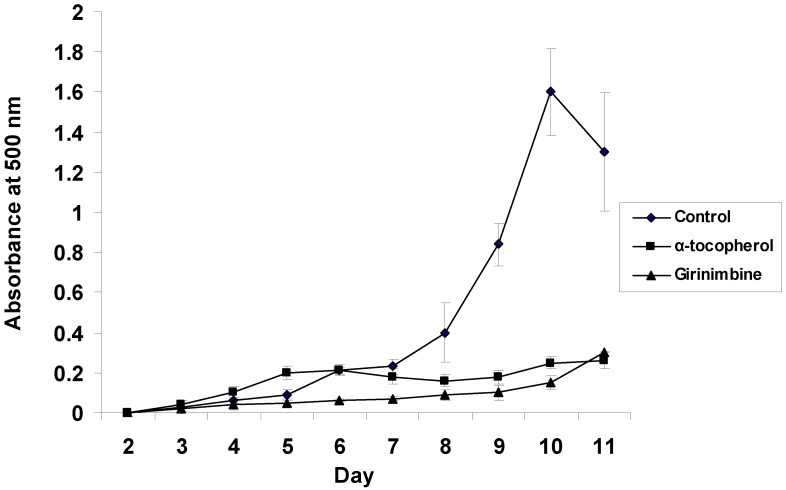
Antioxidant activity of girinimbine measured by FTC method.

**Figure 5:**
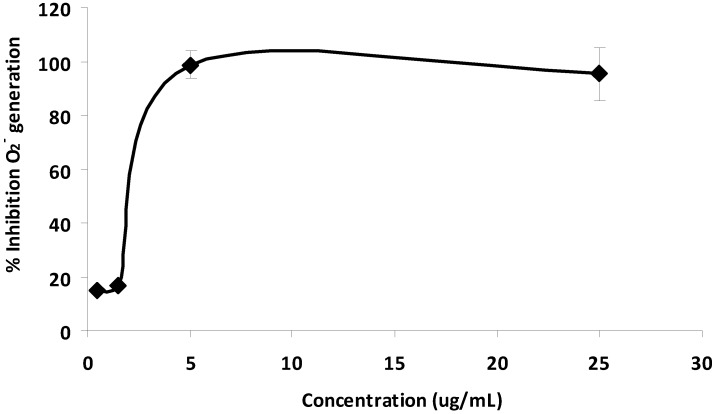
Inhibitory effect of girinimbine on the superoxide generation in differentiated HL60 cells.

Figure 4 shows girinimbine exhibited a very strong antioxidant property which was the same as that vitamin E (α-tocopherol). However, this compound failed to scavenge the diphenyl picryl hydrazyl (DPPH) free radical whereas α-tocopherol did (result not shown). The ability of girinimbine to inhibit the generation of superoxide anion radicals (O_2_^−^) was carried out using the TPA-induced promyelocytic HL-60 cells [24]. Inhibition rate of superoxide anion radicals was at 98.7 and 95.6% when TPA-induced HL-60 cells were treated with girinimbine at the concentrations of 5.3 and 26.3 µg/mL, respectively. However, at concentrations of 0.2 and 1.1 µg/mL the inhibition rate was below 20% (Figure 5). Free radicals have been reported to cause mutagenicity, cytotoxicity and stimulate changes in gene expression. Hydroxyl radicals that form from superoxide anion or hydrogen peroxide are highly active oxygen species which can react directly with DNA, protein and unsaturated lipids of cell. The damaged protein, DNA and lipid peroxidation can cause membrane damage, altered receptor functionality and eventually lead to cell damage. The mechanism of action of girinimbine to inhibit tumour promotion may involve the suppression of O_2_^−^ induced by TPA, since free radicals are reported to participate in altering gene expression during tumour promotion [25].

## 3. Experimental

### 3.1. Isolation of Girinimbine

The stem bark of *M. koenigii* used in this study was collected from Banting, Selangor, Malaysia. Airdried stem bark (1,100 g) was ground into fine powder and extracted by soaking for three days with petroleum ether. The extract was subjected to column chromatography on silica gel eluted with CHCl_3_ to afford girinimbine [26].

### 3.2. Cell Culture

The Raji (human B-lymphoblastoid) cell line was provided by Prof. K. Koshimizu, Kinki University, and premyelocytic HL-60 cell line was obtained from the RIKEN Cell Bank, Tsukuba, Japan. Cells were cultured in RPMI-1640 (Sigma, St. Louis, MO, USA) medium with 10% (v/v) foetal calf serum (Sera Lab, Sussex, UK), 100 IU/mL penicillin (Sigma, St. Louis, MO, USA) and 100 µg/mL streptomycin (Sigma, St. Louis, MO, USA) as a complete growth medium (CGM). Cells were maintained in 25 cm^3^ flask with 10 mL of CGM at 37 °C with 5% CO_2_. Every three days, the cells were sub-cultured by splitting the culture with fresh CGM at a ratio of 2:8. Cell viability was determined using microtetrazolium assay (MTT) as described previously by Mackeen *et al.* [27]. 

### 3.3. Inhibitory Assay of Epstein Barr Virus Activation

The assay was carried out as described by Mooi *et al.* [21]. Briefly, Raji cells at a concentration of 5 × 10^5^ cells/mL were incubated in 1 mL of RPMI 1640 medium (supplemented with 10% fetal calf serum) containing sodium-*n*-butyrate (3 mM), phorbol 12-myristate 13-acetate (0.05 µM) and test substance (5 µL) at 37 °C under 5% CO_2_ for 48 h. Early antigen (EA) expressed in Raji cells was detected by an indirect immunoflourescence method, with EA-positive sera from NPC (nasopharyngeal carcinoma) patients of the Hospital University of the University of Malaya and FITC-labeled IgG (Sigma). The average EA induction was compared to a control with only PMA and sodium-*n*-butyrate, the induction rate of the control was less than 40%.

### 3.4. Immunoblotting Analysis for the Detection of EBV-early Antigen on PVDF Membrane

Immunoblotting of treated and untreated Raji cells was carried out as described by Ali *et al.* [22]. The cells were washed twice with PBS at 4 °C. The cells were spun down at 3,000 g for 5 min at 4 °C and resuspended in five volumes of ice-cold suspension buffer. An equal volume of 2× SDS gel-loading buffer was added, followed by boiling the samples for 10 min. The lysates were centrifuged at 10,000 ×*g* for 10 min. A volume of 15 µL each of the samples and 3 µL of pre-stained SDS-PAGE standards of mid range molecular weight markers (Bio-Rad, Hercules, CA, USA) were loaded into the pre-determined order wells on a 12% SDS-polyacrylamide slab gel [28]. The SDS-polyacrylamide gel containing separated proteins were electrophoretically transferred to a PVDF membrane. The membrane was then stained with NPC serum in the ratio of 1:20, followed by the enzyme-coupled secondary antibody (affinity purified Goat anti Human IgG Horseradish Peroxidase Conjugate (KPL, Gaithersburg, MD, USA)) in the 1:200 ratio with a final concentration of 0.5 µg/mL. The immunoreactive bands in the PVDF membranes were developed with colour development solution consisting of 2% of 4-chloro-1-naphthol (4CN) (Bethesda Research Lab., Gaithersburg, MD, USA) as substrate for Horseradish Peroxidase (HP), 50 mL of Tris HCl buffer, pH 7.4 and 30 µL of 30% of H_2_O_2_.

### 3.5. Antioxidant Assay (Ferric Thiocyanate Method)

The ferric thiocyanate method of antioxidant assay was carried out according to Mohamed *et al.* [29]. Exactly 2 mg of compound was dissolved in of absolute ethanol (4 mL) in a vial with a screw cap (φ 38 × 75 mm). Then, of 2.51% of linolenic acid (4.1 mL, TCI, Tokyo, Japan) in ethanol was added and this was followed by addition of 0.02 M (pH 7) phosphate buffer (8 mL). Lastly, deionised water was added (3.9 mL) and the cap was screw-locked and incubated in an oven at 40 °C in the dark. To an aliquot of this (0.1 mL), 75% ethanol (v/v, 9.7 mL) and 30% ammonium thiocyanate (0.1 mL) were added. Precisely 3 min after addition of 0.02 M ferrous chloride in 3.5% hydrochloric acid (0.1 mL) to the tube, the mixture was shaken and the absorbance was measured at 500 nm wavelength by using a spectrophotometer (Spectronic 20D+, Rochester, NY, USA) for every 24 h until the control reached its maximum absorbance value. 

### 3.6. Diphenyl Picryl Hydrazyl (DPPH) Free Radical Scavenger Assay

Stock solution of the compound at 1 mg/mL was prepared in methanol. The stock solution was diluted to varying concentrations in a volume of 100 µL and was carried out in a 96-well microtiter plate. A volume of 5 µL of diphenyl-*ρ*-picrylhydrazine (DPPH) solution (10 mg/mL in methanol) was added into each well. The plate was kept in the dark for 30 min and the optical density of the wells was measured at a wavelength of 517 nm by using an ELISA Reader (Biotek, Winooski, VT, USA) [29].

### 3.7. TPA-induced Superoxide Generation Test

The assay was performed by using the differentiated human premyelocytic leukemia (HL-60) cells, as described by Nakamura *et al.* [24]. The cells were stimulated to differentiate into granulocyte-like cells by pre-incubation with 1.25% of DMSO in RPMI 1640 medium, supplemented with 10% FBS at 37 °C in 5% CO_2_ incubator for 4 to 6 days. The cells were washed with Hanks buffer (HBSS) and suspended at a density of 1 × 10^6^ cells/mL. Exactly 5 µL of test substance dissolved in DMSO was added into the 1 mL cell suspension and incubated at 37 °C for 15 min. The cells were washed twice with HBSS to remove the extracellular test substance. Ninety sec after stimulation with 12-*O*-tetradecanoylphorbol-13-acetate (TPA) (20 µM), 50 µL of cytochrome C (20 µg/mL) was added to the cell suspension and was incubated for another 15 min at 37 °C. The reaction mixture was stopped by placing it on ice and spun down at 2,000 ×*g* for 1 min. The visible absorption of the supernatant at 550 nm wavelength was determined. The level of O_2_^−^ production was calculated by the following equation (1). The mean value of triplicate was obtained:

[O_2_^−^ (nmol/mL) = 47.7 × A 550 nm] (1) 

All the data obtained the experiments were triplicate and for the cell viability, inhibitory of Epstein-Barr virus the data were expressed as percentage of control.

## 4. Conclusions

Girinimbine, a carbazole alkaloid isolated from the stem bark of *Murraya koenigii* inhibited the expression of early antigen of Epstein Barr virus (EA-EBV) in Raji cells that was induced by tumour promoter, phorbol 12-myristate 13-acetate. The compound was able to suppress restricted early antigen (EA-R) however, diffused early antigen (EA-D) was partially suppressed when used at 32.0 µg/mL. A very strong antioxidant activity was exhibited by girinimbine which was comparable to that of α-tocopherol. The compound was also inhibited the superoxide generation in the TPA-induced differentiated premyelocytic HL-60 cells. 
